# *Paradiplozoon garra* n. sp. (Monogenea, Diplozoidae), a new species from *Garra surgifrons* (Cyprinidae, Labeoninae) in Southwest China

**DOI:** 10.1017/S0031182026102005

**Published:** 2026-05

**Authors:** Lu Shen, Feiyan Meng, Lixian Fan

**Affiliations:** 1 School of Life Science, Yunnan Normal University, Kunming, China; 2 Engineering Research Center for Valorization of Unique Bio-Resources in Yunnan, Ministry of Education, School of Life Sciences, Yunnan Normal University, Kunming, China; 3 Yunnan Key Laboratory of Biomass Energy and Environmental Biotechnology, Yunnan Normal University, Kunming, China

**Keywords:** diplozoids, *Garra surgifrons*, new species, *Paradiplozoon*

## Abstract

In a parasitological survey conducted in 2025 at a fish farm in Kunming, southwest China, a new diplozoid species was discovered on *Garra surgifrons*, representing the first record of a diplozoid previously reported from fish within the genus *Garra* in China. Morphological and molecular evidence confirm that the new species is clearly differentiated from *Paradiplozoon bingolensis*, the only diplozoid previously reported from *Garra* fishes. Phylogenetic analyses place the new species at the basal position of Chinese *Paradiplozoon* species. Considering the natural distribution of its host in Yunnan province and adjacent areas of Myanmar and Laos, southwestern China and Southeast Asia may represent important centres of origin and diversification for East Asian *Paradiplozoon* species. This study highlights the underestimated diversity, host specificity and biogeographic structure of diplozoids in East Asia and underscores the need for expanded sampling in Yunnan and neighbouring Southeast Asian regions.

## Graphical abstract

**Figure:**
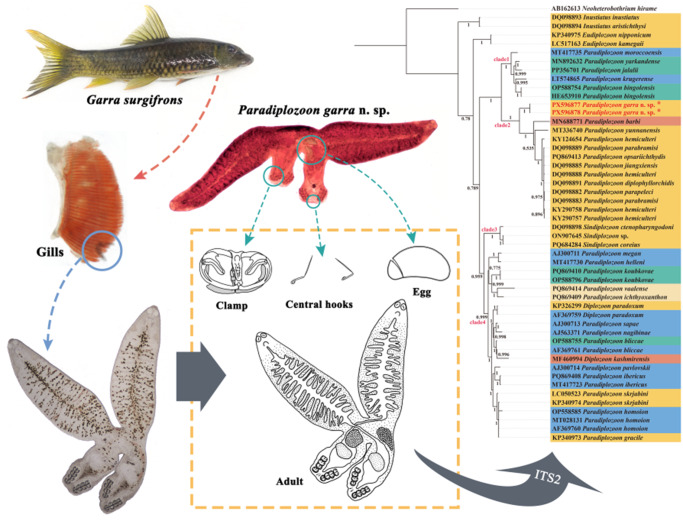

## Introduction

The family Diplozoidae Palombi, 1949 (Monogenea) are highly specialized ectoparasitic flatworms that live almost exclusively on the gills of freshwater cyprinid fishes (Wu et al., 2000; Valigurová et al., 2011; Přikrylová et al., 2018; Benovics et al., 2021). They exhibit a unique life cycle, with life stages including eggs, diporpa, an X-shaped juvenile, and an X-shaped adult (Pečínková et al., 2007a; Avenant-Oldewage and Milne, 2014; Shen et al., 2025).

Like most monogeneans, the taxonomy of diplozoids is based on the sclerotized structures of the haptor, particularly the clamps and central hooks, which provide the primary morphological features for species differentiation (Gussev and Kulemina, 1971; Khotenovsky, 1985; Šebelová et al., 2002; Pečínková et al., 2007b). In the genus *Paradiplozoon* Achmerov, 1974, species identification predominantly relies on the length of the central hooks and the morphology of certain sclerotized elements of the clamps. The combination of the median sclerite and anterior joining sclerite shapes aids in more precise species differentiation (Konstanzová et al., 2017; Jirsová et al., 2018). However, these morphological measurements often exhibit high interspecific similarity and can vary significantly depending on factors such as host species, water temperature, or the geographical origin of the parasites (Matějusová et al., 2002). Additionally, the size and shape of these sclerotized structures may be influenced by fixation and preparation methods, potentially leading to misinterpretation (Huang et al., 2023). Consequently, recent studies have increasingly adopted molecular approaches to complement traditional taxonomy. Among various molecular markers, the internal transcribed spacer 2 (ITS2) region of ribosomal DNA has been widely employed as a genetic marker for species delimitation and phylogenetic analyses of diplozoids. To date, around 60 out of over 80 described species of Diplozoidae have been sequenced for ITS2. While ITS2 has proven useful for species differentiation, it has limitations in distinguishing closely related species, and many originally described species remain unsequenced (Matějusová et al., 2001, 2004; Sicard et al., 2001; Dos Santos and Avenant-Oldewage, 2020).

In recent decades, 9 *Paradiplozoon* species have been described from cyprinid fishes across Africa and Eurasia (Dos Santos et al., 2015; Shimazu et al., 2015; Benovics et al., 2021; Nejat et al., 2023), with 3 species known from China (Fan et al., 2018; Arken et al., 2022; Huang et al., 2023). The diverse freshwater habitats in China support a rich diplozoid fauna. Nevertheless, little is known about diplozoid parasites of the genus *Garra* Hamilton, 1822 (Cyprinidae). To date, only *Paradiplozoon bingolensis* Civáňová, Koyun & Koubková, 2013 has been reported from *Garra rufa* Heckel, 1843 in Turkey (Civáňová et al., 2013), and no species have been recorded from Chinese *Garra* species. The present study reports and describes a new *Paradiplozoon* species from *Garra surgifrons* Zhou and Sun, 2018, a native fish species in China, representing the first record of a diplozoid parasitizing this host genus in China. The type specimen of *G. surgifrons* was collected from the Longchuang River, an upper tributary of the Irrawaddy River drainage in Tengchong, Yunnan (Zhou & Sun, 2018). Further inquiries with aquaculture personnel revealed that the *G. surgifrons* individuals in the farm were introduced from the wild, and the aquaculture water source is drawn from the Irrawaddy River. Morphological characteristics, together with molecular evidence based on the ITS2 region, were employed to confirm its distinct taxonomic status and phylogenetic position within Diplozoidae. Furthermore, the combined morphological, molecular and biogeographic analyses provide new insights into the evolutionary relationships and potential origin of *Paradiplozoon* species in East and Southeast Asia.

## Materials and methods

### Sample collection

In August, September and December 2025, a total of 25 *G. surgifrons* (Cyprinidae, Labeoninae), with a size range of 13–15 cm in standard length, were obtained from a fish farm (102°52’37”N, 25°03’54”E) in Kunming, Yunnan, China. The collected fish were euthanized, and the gills of each fish were subsequently removed and examined using an Olympus SZ-X7 dissecting microscope (Olympus, Japan). The structures of the freshly collected parasite specimens were observed using an Olympus CX41 light microscope (Olympus, Japan) and photographed with the Capture 3.0 software (Tucsen Photonics Co., Ltd). Subsequently, some adult specimens were fixed in 70% ethanol for staining, while the remaining specimens (both larval and adult) were preserved in 95% ethanol for molecular analyses.

### Morphological methods

Specimens preserved in 70% ethanol were rehydrated through a graded ethanol series and soaked in distilled water overnight. Then fixed in Bouin’s solution for 5–7 h, rinsed in distilled water and stained with alum carmine for 12 h. Differentiation was performed in acid alcohol for 20–30 sec, followed by dehydration through a graded ethanol series and clearing in cedarwood oil for 12 h. Finally, specimens were mounted in neutral balsam (Bai et al., 2014). Stained specimens were photographed using an Olympus CX-41 light microscope. Morphometric analyses of parasite structures were conducted using Capture 3.0, and morphological diagrams of the body, clamps, central hooks and eggs were drawn by tracing from existing photographs using a digital drawing board (Wacom, Saitama, Japan, Intuos Pro).

The dimensions of whole body, oral sucker, pharynx, central hooks and clamps characterizing parasites were measured by the straight-line distances between extreme ends. Since the adult body forms an X-shaped fusion body, the body length and width of an individual adult were measured as the straight-line distances between the end-points of the two fused individuals, and the average value was then taken.

### Molecular methods

A total of 7 specimens (3 larvae and 4 adults) were used for molecular analyses, and the remaining specimens were preserved in 95% ethanol. Genomic DNA of the parasites was extracted using the TIANamp Micro DNA Kit (TIANGEN Biotech, Beijing, China) following the manufacturer’s instructions. The ITS2 region of the genomic DNA was amplified using universal primers (Matějusová et al., 2001). Forward primer D: 5′–GGCTYRYGGNGTCGATGAAGAACGCAG–3′; Reverse primer B1: 5′–GCCGGATCCGAATCCTGGTTAGTTTCTTTTCC–3′. Polymerase chain reaction (PCR) was carried out in a 50 μL reaction mixture containing 2 μL DNA template, 19 μL reaction buffer containing dNTPs, 10 × buffer, and Taq polymerase, 2 μL of each primer, and 25 μL double-distilled water. The PCR conditions were as follows: initial denaturation at 90°C for 10 min, followed by 30 cycles of 95 °C for 30 sec for denaturation, 55 °C for 30 sec for annealing and 72 °C for 75 sec for extension, with a final extension at 72 °C for 10 min.

PCR products were verified by 1% agarose gel electrophoresis and stained with GoodView (Tanon, China) to visualize the bands. The amplified DNA fragments were sequenced, and the resulting sequences were submitted to the National Center for Biotechnology Information (NCBI) database and compared using BLAST. A total of 47 diplozoid species sequences were retrieved from NCBI, together with the two sequences obtained in this study, were used for phylogenetic analysis.

Base composition, parsimony analysis and nucleotide substitution rates under p-distance were calculated using MEGA 11.0 (Koichiro et al., 2021). Phylogenetic trees were constructed using both Bayesian Inference (BI) and Maximum Likelihood (ML) methods in Phylosuite v2 (Zhao et al., 2025). The BI analysis was performed with 1 000 000 generations, and ML was conducted using the GTR + G model with 5000 bootstrap replicates. Tree topology robustness was assessed using both BI posterior probabilities and ML bootstrap support. *Neoheterobothrium hirame* Ogawa, 1999 (AB162613) was used as the outgroup for the phylogenetic analysis. The resulting phylogenetic tree was visualized using the Interactive Tree of Life (iTOL) platform (Letunic and Bork, 2021), with the aid of dataset files generated by Phylosuite v2.

## Results

Family: Diplozoidae Palombi, 1949

Genus: *Paradiplozoon* Achmerov, 1974

Type species: *Paradiplozoon garra* n. sp.

ZooBank LSID: urn:lsid:zoobank.org:pub:5CCFCE3B-C6A0-44F6-9232-DB60A36FEF48

**Figure 1.**
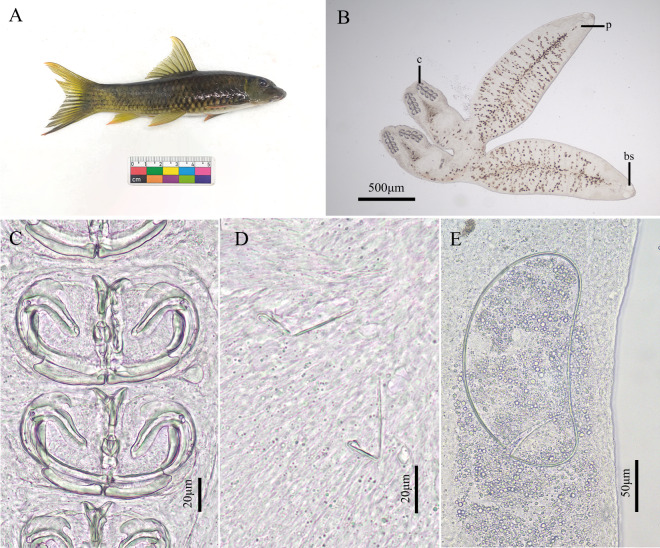
Photographs of host fish and *Paradiplozoon garra* n. sp. (A) *Garra surgifrons* Zhou & Sun, 2018; (B) whole body of worm; (C) clamps; (D) central hooks; (E) egg. (bs, buccal suckers; p, pharynx; c, clamps). Figure 1 long description.

Type-host: *G. surgifrons* Zhou & Sun, 2018 (Cyprinidae, Labeoninae) (Figure 1A)

Site: Gills

Infection intensity: 1–2 parasites per infected fish

Infection rate: 1.60, 32 parasites in 20 fish

Type locality: a fish farm in Kunming, Yunnan, China. (102°52’37”N, 25°03’54”E)

Type material: Holotype (H2025090401), 6 paratypes (H2025090402, H2025090403, H2025090404, H2025080505, H2025120306, H2025120307), deposited in the Museum of Yunnan Normal University, Yunnan, China.

Etymology: The specific name *garra* refers to the host genus *G. surgifrons*.

### Description

Description based on observation of 11 adult specimens. Body surfaces smooth, without folds. Anterior region of buccal suckers lacks round glands. No disc-shaped widened area with disciform structure between posterior and reproductive fusion areas, nor any special lobed enlargement (Figures 1B and 3A).

**Figure 2.**
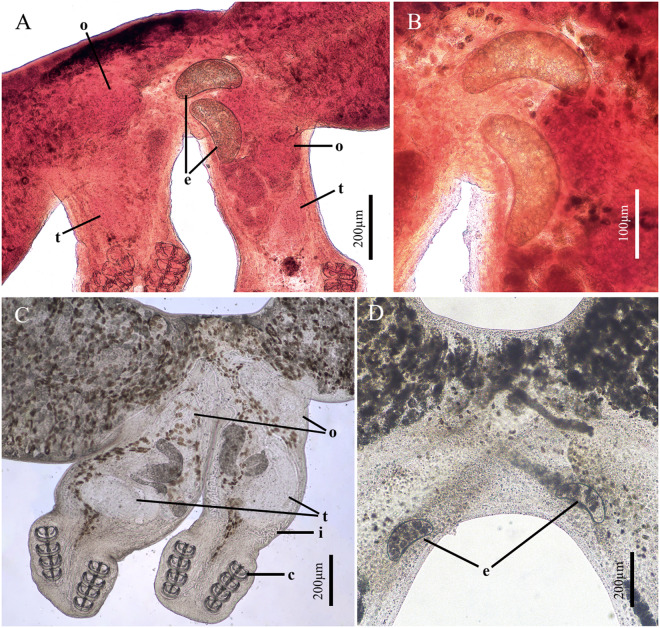
Reproductive system of *Paradiplozoon garra* n. sp. (A) Reproductive system; (B) The eggs; (C) Reproductive system without eggs; (D) Reproductive system with eggs. (o, ovary; t, testis; e, eggs; i, intestine; c, clamps). A and B show stained specimens; C and D show specimens mounted in water. Figure 2 long description.

Adult specimens measure 4.610 (2.373–6.941, *n* = 8) mm in length and 0.724 (0.520–0.955, *n* = 8) mm in width. Intestine located centrally, extending to body fusion area. Vitelline follicles numerous, distributed at anterior part of body. Reproductive glands extend from fusion area to posterior part of body. Ovary single, 0.286 (0.253–0.335, *n* = 3) × 0.206 (0.134–0.283, *n* = 3) mm, situated in fusion area. Testis single, 0.223 (0.187–0.315, *n* = 3) × 0.189 (0.149–0.232, *n* = 3) mm, compact, unlobed, positioned posterior to ovary (Figures 2A and C).

**Figure 3.**
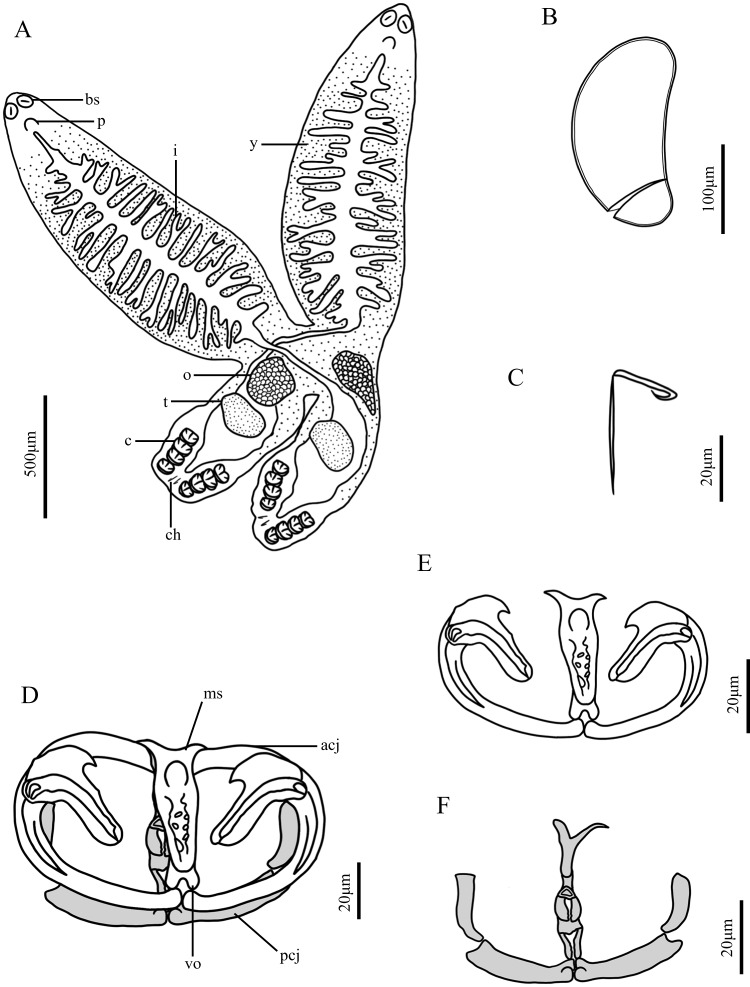
Digital drawing of *Paradiplozoon garra* n. sp. (A) Adult; (B) egg; (C) Central hooks; (D) Clamp; (E) Clamp in anterior view; (F) Clamp in posterior view. (bs, buccal suckers; p, pharynx; i, intestine; y, vitellarium; o, ovary; t, testis; c, clamps ch, central hooks; ms, median sclerite; acj, anterior clamp jaw; pcj, posterior clamp jaw; vo, V-outgrowth). Figure 3 long description.

Eggs elliptical, lacking filaments attached to operculum (Figures 1E, 2B, 2D and 3B), 0.101 (0.094–0.110, *n* = 3) × 0.213 (0.205–0.221, *n* = 3) mm. Haptors measure 0.324 (0.258–0.369, *n* = 8) mm in length and 0.387 (0.316–0.493, *n* = 8) mm in width, bear four pairs of clamps and one pair of central hooks. Clamp I smallest, 0.062 (0.041–0.077, *n* = 15) × 0.090 (0.065–0.124, *n* = 15) mm. Clamp II 0.064 (0.050–0.080, *n* = 15) × 0.103 (0.077–0.130, *n* = 15) mm. Clamp III largest, 0.066 (0.053–0.079, *n* = 16) × 0.104 (0.074–0.127, *n* = 16) mm. Clamp IV 0.064 (0.052–0.075, *n* = 16) × 0.097 (0.068–0.124, *n* = 16) mm. Anterior clamp jaw composed of two curved sclerites. Posterior clamp jaw consists of medial and lateral parts. Both anterior arch of anterior clamp jaw and medial part of posterior jaw lack cross-striation. Spurs of anterior jaws form thin, elongated, hook-shaped structures. Anterior half of median plate shows thickened margins with irregularly arranged circular perforations; anterior joining sclerites short and V-shaped. Posterior half of median plate presents irregular, short trapezoid-like spur and pair of elongated, rod-shaped joining sclerites (Figures 1C and 3D–F).

Central hooks positioned between terminal protrusion of haptor and first pair of clamps. Hooks formed by handle and sickle connected together. Sickle curves toward handle, ending in winged tip curling toward connection. Central hook sickle 0.016 (0.012–0.021, *n* = 15) mm, handle 0.037 (0.029–0.045, *n* = 15) mm (Figures 1D and 3C).

### Diagnosis

The new diplozoid species described from *G. surgifrons* differs markedly from *P. bingolensis*, collected from another congeneric host (*G. rufa*), in both morphological characteristics and molecular sequences. The main distinguishing features of *P. bingolensis* include: a rectangular anterior joining sclerite connected to the proximal tip of the anterior jaw. The posterior end of the median plate narrowing and terminating with a wide, rounded sclerite with an opening, and has a short posterior joining sclerite. In contrast, *P. garra* is characterized by thin, elongated, hook-shaped spurs on the anterior jaws. The anterior half of the median plate bears short V-shaped joining sclerites, while the posterior half features an irregular, short trapezoid-like spur with a pair of elongated, rod-shaped joining sclerites. Morphometric comparisons of *P. garra* with other closely related species of the genus are summarized in Table 1.

**Table 1.** Measurements of *Paradiplozoon garra* n. sp. and closely related *Paradiplozoon* species Table 1 long description.

	*Paradiplozoon garra* n. sp.	*P. bingolensis*	*P. yunnanese*	*P. cirrhini*	*P. hemiculteri* (Ling, 1973)	*P. yarkandense*
Host	*Garra surgifrons*	*Garra rufa*	*Sikukia gudgeri*	*Cirrhinus molitorella*	*Hemiculter leucisculus*	*Schizothorax eurystomus*
Body length	4610 (2373–6941)	3850 (3200– 4500)	1593 (1148–2344)	2813 (1376–6314)	3153 (2320–4180)	2130 (1100–3050)
Body width	724 (520–955)	−	445 (211–626)	668 (443–1107)	931 (729–1140)	690 (440–1010)
Ovary	286 (253–335) × 206 (134–283)	−	−	−	−	−
Testis	223 (187–315) × 189 (149–232)	−	−	−	−	−
Haptor length	324 (258–369)	−	−	268 (202–344)	−	340 (240–510)
Haptor width	387 (316–493)	−	−	428 (308–657)	−	300 (180–410)
Oral sucker	80 (66–102) × 67 (47–94)	−	46 (23–65) × 35 (26–44)	71 (41–110) × 78 (44–115)	58 (50–67) × 54 (42–65)	60 (50–80) × 50 (40–70)
Pharynx	75 (62 − 94) × 61 (44–86)	−	45 (30 − 56) × 38 (21 − 52)	52 (38 − 64) × 70 (58 − 94)	66 (50 − 83) × 54 (38 − 62)	47 (46 − 49) × 42 (41 − 43)
Clamp I	62 (41–77) × 90 (65–124)	144.7 − 153.3 × 108.5 − 114.6	39 (18–59) × 53 (23–78)	60 (31–105) × 96 (61–142)	56 (42–70) × 90 (72 − 119)	70 (50–80) × 110 (90–120)
Clamp II	64 (50–80) × 103 (77–130)	143.8–146.3 × 101.2–107.4	44 (20–65) × 61 (27–86)	65 (33–104) × 120 (74–187)	60 (49–69) × 104 (91–115)	90 (80–100) × 150 (140–180)
Clamp III	66 (53–79) × 104 (74–127)	150.5–151.4 × 97.9–101.3	43 (17–61) × 62 (29–89)	67 (36–112) × 127 (83–215)	61 (47–82) × 107 (74–127)	100 (90–110) × 160 (140–190)
Clamp IV	64 (52–75) × 97 (68–124)	144.7–153.2 × 108.5–114.6	46 (20–59) × 62 (27–87)	73 (41–119) × 126 (81 − 195)	62 (47–82) × 105 (76–126)	80 (70–90) × 140 (120–170)
Central hook sickle	16 (12–21)	17.5–20.8	12	14 (11–18)	17.6 (17–18.1)	23 (19–26)
Central hook handle	37 (29–45)	−	21.5	36 (26–41)	36.6 (34–37.9)	42 (39–45)
Egg	213 (205–221) × 101 (94–110)	−	151 (142–160) × 75 (74–87)	333 (327–338) × 151 (133–170)	−	200 (180–220) × 70 (60–80)
References	Present study	Civáňová et al., 2013	Fan et al., 2018	Huang et al., 2023	Jirsová et al., 2018	Arken et al., 2022

The values of measurement in micrometre (µm).

### Molecular analyses

Sequence comparison of the ITS2 region from all seven specimens revealed identical sequences. Two representative specimens, one adult and one larva, were selected for subsequent ITS2 phylogenetic analysis, yielding sequences of 794 bp and 740 bp, respectively. The resulting sequences were submitted to GenBank under accession numbers PX596877 and PX596878. A total of 47 representative ITS2 sequences of diplozoid species were selected from different geographical regions, with detailed species information provided in Table 2. Alignment of the 49 ITS2 sequences yielded a total sequence length of 738 bp, comprising 215 conserved sites (29.1%), 523 variable sites (70.9%) and 433 parsimony-informative sites (58.7%). The relatively high proportion of variable and parsimony-informative sites indicates substantial sequence divergence among taxa.

**Table 2.** List of diplozoid species, host species, locality of collection and GenBank accession numbers for DNA sequences used in phylogenetic analyses Table 2 long description.

Parasite species	Host species	Locality	Length (bp)	Accession number	Reference
*Inustiatus inustiatus* (Nagibina, 1965) Khotenovsky, 1978	*Hypophthalmichthys molitrix*	Tangxun Lake, China	771	DQ098893	Gao et al., 2007
*I. aristichthysi* (Ling, 1973) Jiang, Wu & Wang, 1989	*Hypophthalmichthys nobilis*	Tangxun Lake, China	771	DQ098894	Gao et al., 2007
*Eudiplozoon nipponicum* Goto, 1891	*Cyprinus carpio*	Irtysh River, China	772	KP340975	N/A
*Eudiplozoon kamegaii* Nishihira and Urabe, 2020	*Cyprinus carpio*	Shiga Prefecture, Japan	625	LC517163	Nishihira et al., 2020
*Paradiplozoon moroccoensis* Benovics et al., 2021	*Barbus lepineyi*	Zoula Oasis, Morocco	671	MT417735	Benovics et al., 2021
*P. yarkandense* Arken et al., 2022	*Schizothorax eurystomus*	Yarkand River, Xinjiang, China	807	MN892632	Arken et al., 2022
*P. jalalii* Shamsi, et al., 2025	*Schizocypris altidorsalis*	Iran	774	PP356701	Shamsi et al., 2024
*P. krugerense* Dos Santos & Avenant-Oldewage, 2016	*Labeo rosae*	Olifants River, South Africa	757	LT574865	Dos Santos and Avenant-Oldewage, 2020
*P. bingolensis* Civáňová et al., 2013	*Cyprinion macrostomum*	Iraq	773	OP588754	Nejat et al., 2023
*P. bingolensis* Civáňová et al., 2013	*Garra rufa*	Turkey	725	HE653910	Civáňová et al., 2013
*P. yunnanense* Fan et al., 2018	*Sikukia stejnegeri*	China	853	MT336740	Fan et al., 2018
*P. hemiculteri* (Ling, 1973) Khotenovsky, 1985	*Hemiculter leucisculus*	China	820	KY124654	Jirsová et al., 2018
*P. parabramisi* (Ling, 1973) Khotenovsky, 1985	*Parabramis pekinensis*	China	821	DQ098889	Gao et al., 2007
*P. opsariichthydis* (Jiang, Wu & Wang, 1984) Jiang, Wu & Wang, 1989	*Opsariichthys bidens*	China	717	PQ869413	Jirsová et al., 2021
*P. jiangxiense* (Jiang, Wu & Wang, 1985) Jiang, Wu & Wang, 1989	*Chanodichthys erythropterus*	Tangxun Lake, China	822	DQ098885	Gao et al., 2007
*P. hemiculteri* (Ling, 1973) Khotenovsky, 1985	*Hemiculter leucisculus*	Danjiangkou Reservoir, China	822	DQ098888	Gao et al., 2007
*P. diplophyllorchidis* (Jiang, et al., 1985) Jiang, Wu & Wang, 1989	*Zacco platypus*	Danjiangkou Reservoir, China	821	DQ098891	Gao et al., 2007
*P. hemiculteri* (Ling, 1973) Khotenovsky, 1985	*Hemiculter leucisculus*	Honghu, China	822	DQ098882	Gao et al., 2007
*P. parabramisi* (Ling, 1973) Khotenovsky, 1985	*Parabramis pekinensis*	Tangxun Lake, China	822	DQ098883	Gao et al., 2007
*P. hemiculteri* (Ling, 1973) Khotenovsky, 1985	*Hemiculter leucisculus*	Wuxi, China	1762	KY290758	Jirsová et al., 2018
*P. hemiculteri* (Ling, 1973) Khotenovsky, 1985	*Hemiculter leucisculus*	Wuxi, China	1762	KY290757	Jirsová et al., 2018
*S. ctenopharyngodoni* (Ling, 1973) Jiang, Wu & Wang, 1989	*Ctenopharyngodon idella*	Tangxun Lake, China	834	DQ098898	Gao et al., 2007
*Sindiplozoon* sp.	*Squaliobarbus curriculus*	China	596	ON907645	Huang et al., 2023
*Sindiplozoon coreius* Cao et al., 2021	*Chanodichthys erythropterus*	Lancang River, China	829	PQ684284	Shen et al., 2025
*P. megan* Bychowsky & Nagibina, 1959	*Leuciscus cephalus*	Morava River, Czech Republic	774	AJ300711	Matějusová et al., 2001
*P. helleni* Benovics et al., 2021	*Tropidophoxinellus hellenicus*	Greece	652	MT417730	Benovics et al., 2021
*P. koubkovae* Řehulková, Nejat & Benovics, 2023	*Luciobarbus capito*	Iran	762	PQ869410	Benovics et al., 2025
*P. koubkovae* Řehulková, Nejat & Benovics, 2023	*Capoeta kaput*	Turkey	744	OP588796	Nejat et al., 2023
*P. vaalense* Dos Santos, Jansenvan Vuuren & Avenant-Oldewage, 2015	*Labeo umbratus*	South Africa	700	PQ869414	Benovics et al., 2025
*P. ichthyoxanthon* Avenant-Oldewage and Milne, 2013	*Labeobarbus aeneus*	South Africa	718	PQ869409	Benovics et al., 2025
*Diplozoon paradoxum* Nordmann, 1832	*Abramis brama*	China	771	KP326299	N/A
*D. paradoxum* Nordmann, 1832	*Abramis brama*	France	1002	AF369759	Sicard et al., 2001
*P. sapae* Reichenbach-Klinke, 1961	*Blicca bjoerkna*	Morava River, Czech Republic	769	AJ300713	Matějusová et al., 2001
*P. nagibinae* (Gläser, 1965) Khotenovsky, 1985	*Ballerus ballerus*	Kyjovka River, Czech Republic	761	AJ563371	Matějusová et al., 2004
*P. bliccae* Reichenbach-Klinke, 1961	*Barbus xanthos*	Turkey	784	OP588755	Nejat et al., 2023
*D. bliccae* Reichenbach-Klinke, 1961	*Blicca bjoerkna*	France	988	AF369761	Sicard et al., 2001
*D. kashmirensis* Kaw, 1950	N/A	India	848	MF460994	N/A
*P. pavlovskii* (Bychowsky & Nagibina, 1959) Khotenovsky, 1985	*Leuciscus aspius*	Morava River, Czech Republic	770	AJ300714	Matějusová et al., 2001
*P. ibericus* Benovics et al., 2021	*Squalius valentinus*	Spain	643	MT417723	Benovics et al., 2021
*P. ibericus* Benovics et al., 2021	*Iberochondrostoma lusitanicum*	Portugal	651	PQ869408	Benovics et al., 2025
*P.skrjabini* (Akhmerov, 1974) Khotenovsky, 1985	*Tribolodon hakonensis*	Tenryu River, Japan	683	LC050523	Shimazu et al., 2015
*P. skrjabini* (Akhmerov, 1974) Khotenovsky, 1985	*Leuciscus baicalensis*	China	764	KP340974	N/A
*P. homoion* (Bychowsky & Nagibina, 1959) Khotenovsky, 1985	*Barbus oligolepis*	Turkey	808	OP558585	Aydoğdu and Torcu Koç, 2023
*P. homoion* (Bychowsky & Nagibina, 1959) Khotenovsky, 1985	*Rhodeus amarus*	Turkey	820	MT028131	
*D. homoion* Bychowsky & Nagibina, 1959	*Rutilus rutilus*	France	996	AF369760	Sicard et al., 2001
*P. gracile* (Reichenbach Klinke, 1961)	*Gobio acutipinnatus*	Irtysh River (Chinese section), China	764	KP340973	N/A
*Paradiplozoon garra* n. sp.	*Garra surgifrons*	Yunnan, China	794	PX596877	Present study
*Paradiplozoon garra* n. sp.	*Garra surgifrons*	Yunnan, China	740	PX596878	Present study

BLAST analysis showed that, when compared with published ITS2 sequences of diplozoid species, sequence homology ranged from 78.20% to 81.36% within 98–100% coverage. The highest similarity was observed with *Paradiplozoon parabramisi* (Ling, 1973) Khotenovsky, 1985 (DQ098883) and *P. opsariichthydis* (Jiang, Wu & Wang, 1984) Jiang, Wu & Wang, 1989 (DQ098890), both exhibiting 81.36% similarity. The genetic p-distance (Supplementary materials) between *P. garra* n. sp. and other diplozoid species ranged from 16.42% to 33.68%. The species most closely related to *P. garra* n. sp. were *P. hemiculteri* (Ling, 1973) Khotenovsky, 1985 (KY124654), *P. parapeleci* Jiang et al., 1984 (DQ098882), and *P. parabramisi* (DQ098883), with genetic p-distance of 16.42%, 16.55% and 16.55%, respectively (see Supplementary materials).

The phylogenetic tree showed that within the Diplozoidae, species of the genus *Inustiatus* Khotenovsky, 1978 diverged first, followed by the genus *Eudiplozoon* Khotenovsky, 1985. The remaining genera, *Paradiplozoon, Sindiplozoon* Khotenovsky, 1981, and *Diplozoon* von Nordmann, 1832, formed a large clade that was sister to *Eudiplozoon*. This large clade was further subdivided into two distinct subclades. Within one of the subclades, the two sequences of the newly described species (*P. garra* n. sp. PX596877, PX596878) formed a highly supported monophyletic group and clustered with other *Paradiplozoon* species from China forming clade 2. Clade 2 was sister to clade 1, which comprised *Paradiplozoon* species from the Middle East, China, Europe and South Africa, including *P. krugerense* Dos Santos & Avenant-Oldewage, 2016. In the other subclade, three *Sindiplozoon* species (DQ098898, ON907645, PQ684284) from China formed a highly supported group, designated as clade 3.

Clade 4 was sister to clade 3 and included *Paradiplozoon* and *Diplozoon* species from Europe, *Paradiplozoon* species from the Middle East, South Africa, China and Japan, as well as *Diplozoon paradoxum* von Nordmann, 1832 (KP326299) from China and *D. kashmirensis* Kaw, 1950 (MF460994) from India. While *D. kashmirensis* from India (MF460994) clustered with *P. bliccae* Reichenbach-Klinke, 1961 (AF369761) from France.

## Discussion

Members of the genus *Paradiplozoon* exhibit distinctive morphological traits, making their identification at the genus level relatively straightforward. However, species-level identification remains challenging. In the present study, an integrative approach combining morphological and molecular analyses led to the identification of a new species, *Paradiplozoon garra*, collected from the gills of *G. surgifrons* in Kunming, southwest China.

In November 2024, a large number of *Sindiplozoon coreius* Cao, 2022 (Shen et al., 2025) were collected from cultured *Anabarilius grahami* Regan, 1908 at the same fish farm where *G. surgifrons* samples were obtained. Notably, *G. surgifrons* was not co-cultured with *A. graham* and originated from a different source (with *A. grahami* introduced from Dali, Yunnan, and *G. surgifrons* from the Irrawaddy River), indicating that the farming conditions at this site are highly conducive to diplozoids proliferation, potentially detrimental to fish health. Other fish species co-cultured with *G. surgifrons*, including *Barbodes hexagonolepis* McClelland, 1839, *Phoxinus lagowskii* Dybowski, 1869 and *Hemibarbus maculatus* Bleeker, 1871, showed no signs of diplozoids infection, suggesting that *P. garra* exhibits a certain level of host specificity.

Morphologically, *P. garra* produces eggs without attached filaments, which reduces the likelihood that its eggs can remain stably adhered to gill filaments after being laid. Its attachment structures (central hooks and clamps) are relatively small compared to related species (Table 1), which is unfavourable for attachment. While the pronounced spurs on the clamps, distinct from other *Paradiplozoon* species, may enhance attachment efficiency. These morphological features, particularly the small size of central hooks and clamps combined with the unique spur on the clamps and the filament-free eggs, distinguish *P. garra* from all other known *Paradiplozoon* species and support its recognition as a new species. These observations suggest that its parasitic ability may be relatively unaffected by environmental conditions, possibly adapting to slower-flowing natural waters or resource-rich aquaculture environments, and adopting a relatively gentle attachment strategy. However, no wild populations of *P. garra* have yet been found, and future studies are needed to verify these hypotheses regarding its morphology and survival strategies.

**Figure 4.**
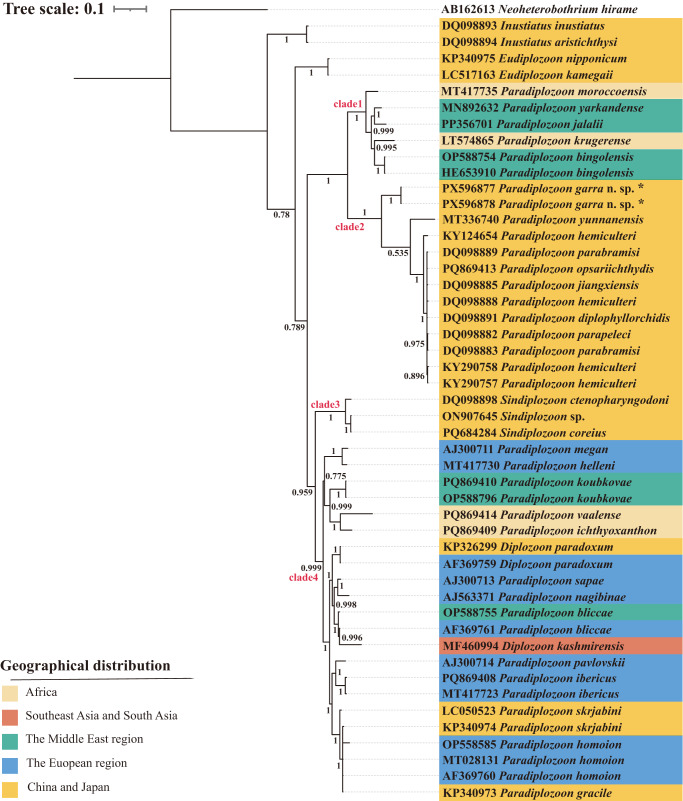
The rooted condensed tree of the parasite geographical distribution based on the BI analysis of ITS2 sequences of diplozoids. The numbers at nodes indicate posterior probabilities (%). The monogenean *Neoheterobothrium hirame* Ogawa, 1999 was used as the outgroup. Species marked with an * in the figures represent sequences from this study. Figure 4 long description.

**Figure 5.**
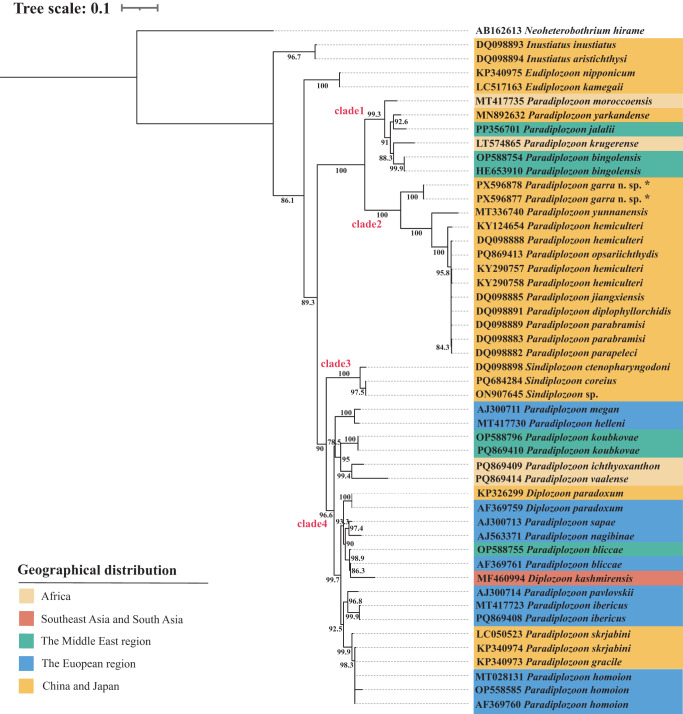
The rooted condensed tree of the parasite geographical distribution based on the ML analysis of ITS2 sequences of diplozoids. The numbers at nodes indicate bootstrap values (%). The monogenean *Neoheterobothrium hirame* Ogawa, 1999 was used as the outgroup. Species marked with an * in the figures represent sequences from this study. Figure 5 long description.

Phylogenetic analyses (Figures 4 and 5), together with previous studies (Huang et al., 2023), indicate that Chinese *Paradiplozoon* species belong to a relatively derived clade within Diplozoidae (Clade 2). Within this clade, *P. yunnanense* (Fan et al., 2018; GenBank accession no. MT336740) from Yunnan occupies a basal position among Chinese taxa. Based on these findings, we speculate that *Paradiplozoon* populations distributed in southwestern China and extending into Southeast Asia may represent more evolutionarily primitive lineages, suggesting that Southeast Asia could be a potential origin centre for *Paradiplozoon* in East Asia. The newly described species in this study forms a distinct branch with high support (posterior probability = 1). It occupies a basal branch within Clade 2 and may represent a relatively ancestral lineage. Considering the natural distribution of its host in Yunnan (Lianghe County, Mengyang Town; Tengchong City, Mangbang Town) (Sun et al., 2018), it is reasonable to infer that natural populations of this parasite may also occur in adjacent regions of Myanmar and Laos, consistent with the geographic pattern shown in the phylogenetic tree. Compared with its close relatives in Clade 2, *P. garra* differs in host species (representing the first record of a diplozoid infecting a *Garra* species in China), geographic distribution (potentially occurring in the Irrawaddy River basin), and key morphological characters such as clamp and egg morphology, reinforcing the phylogenetic distinctions observed in our ITS2 analyses. Combined with the evidence, the species in this study is recognized as a new species. However, due to limited research on diplozoid parasites in Yunnan and Southeast Asia (Bai et al., 2014; Fan et al., 2018), current evidence is insufficient to fully support this hypothesis regarding their geographic distribution and origin, and further data are needed for validation.

The phylogenetic tree also shows that *P. yarkandense* from China clusters with *P. moroccoensis* from Morocco, along with several diplozoid species from the Middle East and Europe, forming Clade 1. This clustering supports the hypothesis that *P. yarkandense*, distributed in the Yarkand River in northwest China (Xinjiang), is geographically and phylogenetically close to Middle Eastern diplozoids. Similarly, *P. moroccoensis* from North Africa, near Europe, clusters with European diplozoid species.

While ITS2 is widely applied for species identification across monogeneans, including *Paradiplozoon*, its resolution for distinguishing closely related species is limited, and it may not accurately reflect deeper phylogenetic relationships. Therefore, ITS2 should ideally be complemented with additional markers and morphological analyses to achieve a robust understanding of species diversity.

In conclusion, this study improves our understanding of species diversity, host specificity and phylogenetic relationships within Diplozoidae. The discovery of *P. garra* from *G. surgifrons* represents the first record of a diplozoid infecting this host genus in China, indicating that diplozoid diversity in the region is likely greater than currently known. Phylogenetic analyses suggest that Chinese *Paradiplozoon* species form a derived lineage, with *P. garra* and *P. yunnanensis* occupying the base of this lineage, implying that southwestern China may be an important centre of origin and diversification for these parasites. Future studies should expand sampling across Yunnan and neighbouring Southeast Asian regions to further elucidate the evolutionary history and biogeographic patterns of diplozoid parasites. Moreover, in high-density, resource-rich aquaculture environments, diplozoids can proliferate rapidly, causing significant fish mortality, highlighting the urgent need for effective management strategies to mitigate infection risks.

## Supporting information

10.1017/S0031182026102005.sm001 Shen et al. supplementary material Shen et al. supplementary material

## Supplementary material

The supplementary material for this article can be found at https://doi.org/10.1017/S0031182026102005.

## Figure 1 Long description

The image A showing a fish in side view on a white background, with a rectangular multicolor measurement scale labeled cm below the fish. The image B showing a pale, branched structure on a light background with many small dark dots. Three label lines mark c, p and bs. A scale bar reads 500µm. The image C showing a micrograph with repeated paired curved structures arranged in rows on a pale background. A vertical scale bar reads 20µm. The image D showing a micrograph with a pale background containing many fine, elongated streak-like elements and two thin, angled line-like structures near the center. A vertical scale bar reads 20µm. The image E showing a micrograph with an oval outlined structure containing many small circular elements on a pale background. A vertical scale bar reads 50µm.

Navigate back to Figure 1.

## Figure 2 Long description

The image A showing a red-stained tissue section with a large white curved space near the center. Three black label lines point to areas marked with the letters “o”, “e” and “t”. A vertical scale bar at the right reads “200µm”. The image B showing a red-stained tissue section with a large white curved space near the lower left. A vertical scale bar at the right reads “100µm”. The image C showing a gray-stained tissue section with two rounded projections at the lower left and lower center, each containing multiple small oval structures. Black label lines point to areas marked with the letters “o”, “t”, “i” and “c”. A vertical scale bar at the right reads “200µm”. The image D showing a gray-stained tissue section with a large white curved space along the bottom. A black label line points to an area marked with the letter “e”.

Navigate back to Figure 2.

## Figure 3 Long description

Insufficient visual information to describe this element accurately.

Navigate back to Figure 3.

## Figure 4 Long description

The image presents a rooted phylogenetic tree illustrating the geographical distribution of diplozoids based on Bayesian Inference analysis of ITS2 sequences. The tree is oriented vertically, with branches extending from a single root at the top. The tree scale is indicated as 0.1. The tree is divided into four main clades labeled as clade 1, clade 2, clade 3 and clade 4, each marked in red. Posterior probabilities are shown at various nodes, indicating the statistical support for each branch. The species names are listed on the right, each associated with a specific geographical region indicated by color: Africa (orange), Southeast Asia and South Asia (green), the Middle East region (red), the European region (blue) and China and Japan (yellow). The outgroup used is Neoheterobothrium hirame. Clade 1 includes species such as Inustiatus aristichthydis and Paradiplozoon jalalii. Clade 2 features species like Paradiplozoon yunnanense and Paradiplozoon garra n. sp., with some sequences marked with an asterisk indicating they are from the current study. Clade 3 includes species such as Paradiplozoon megan and Paradiplozoon paradoxum. Clade 4 contains species like Paradiplozoon bliccae and Paradiplozoon skrjabini. The geographical distribution legend is located at the bottom left, explaining the color coding for each region.

Navigate back to Figure 4.

## Figure 5 Long description

The image displays a rooted phylogenetic tree illustrating the geographical distribution of diplozoid parasites based on ITS2 sequence analysis. The tree is oriented vertically, with branches extending from the top to the bottom. The tree scale is indicated as 0.1 at the top left. The tree begins with Neoheterobothrium hirame as the outgroup, followed by several clades labeled clade 1, clade 2, clade 3 and clade 4. Each clade is marked with bootstrap values at the nodes, indicating the statistical support for each branch. Clade 1 includes species such as Paradiplozoon yarkandense and Paradiplozoon jalalii, with bootstrap values ranging from 99.3 to 100. Clade 2 features species like Paradiplozoon yunnanense and Paradiplozoon hemiculteri, with bootstrap values from 84.3 to 100. Clade 3 contains species such as Paradiplozoon megan and Paradiplozoon helleni, with bootstrap values from 75 to 100. Clade 4 includes species like Diplozoon paradoxum and Diplozoon bliccae, with bootstrap values from 96.6 to 100. The geographical distribution is indicated by color coding: Africa in beige, Southeast Asia and South Asia in red, the Middle East region in teal, the European region in blue and China and Japan in yellow. Species marked with an asterisk represent sequences from the study. The tree visually represents the evolutionary relationships and geographical distribution of these parasites.

Navigate back to Figure 5.

## Table 1 Long description

The table compares micrometre-scale measurements for Paradiplozoon garra n. sp. and five related Paradiplozoon species, alongside their fish hosts. Measurements include body length and width, reproductive organs (ovary and testis where available), haptor size, oral sucker and pharynx dimensions, four clamp sizes, central hook parts, and egg size, with values typically given as an average followed by a range. Paradiplozoon garra n. sp. from Garra surgifrons has the greatest mean body length at 4610 micrometres, compared with 3850 for P. bingolensis, 3153 for P. hemiculteri, 2813 for P. cirrhini, 2130 for P. yarkandense, and 1593 for P. yunnanese. Body width is reported for most species and is highest in P. hemiculteri at 931 micrometres; P. bingolensis lacks a body-width value. Haptor measurements are incomplete across species, but where reported P. yarkandense has a longer mean haptor length at 340 micrometres than P. garra at 324 and P. cirrhini at 268, while P. cirrhini shows a wider mean haptor width at 428 micrometres than P. garra at 387 and P. yarkandense at 300. Clamp dimensions show a strong contrast: P. bingolensis has much larger clamps, with lengths around 145 to 153 micrometres, whereas the other species generally have clamp lengths around 39 to 100 micrometres depending on clamp number and species. Central hook sickle length is greatest in P. yarkandense at 23 micrometres, while P. yunnanese is smallest at 12; central hook handle values are missing for P. bingolensis. Egg size varies widely, with P. cirrhini having the largest eggs at 333 by 151 micrometres, compared with 213 by 101 in P. garra and 151 by 75 in P. yunnanese; egg data are not provided for P. bingolensis or P. hemiculteri. Several cells contain dashes indicating unreported measurements, so comparisons should be interpreted as limited to the available data and may reflect differences in reporting across sources.

Navigate back to Table 1.

## Table 2 Long description

The table lists diplozoid parasite taxa used for phylogenetic work and pairs each record with its fish host, collection locality, DNA sequence length in base pairs, GenBank accession number, and literature source. Most entries are Paradiplozoon species, with additional records for Inustiatus, Eudiplozoon, Sindiplozoon, and Diplozoon. Localities span Asia, Europe, and Africa, but China dominates the sampling, including multiple sites such as Tangxun Lake, Danjiangkou Reservoir, the Irtysh River, and Yunnan. Several parasites appear in multiple records across different places or hosts, for example Paradiplozoon hemiculteri is listed from several Chinese localities and includes two notably longer sequences from Wuxi, while Diplozoon paradoxum and related taxa include records from both China and France. Sequence lengths vary widely, from under six hundred base pairs to over one thousand seven hundred base pairs, indicating that different fragments or sequencing strategies were used across studies. Some rows have missing information, such as an unspecified host for Diplozoon kashmirensis and missing or not available references for a few accessions. Two entries labeled as a new Paradiplozoon garra species from Garra surgifrons in Yunnan are attributed to the present study and provide two accessions with different sequence lengths. Because the dataset compiles sequences from multiple publications and regions, differences in length and completeness should be considered when comparing records.

Navigate back to Table 2.
